# Epidermal Growth Factor Receptor–Targeted Fluorescence Molecular Imaging for Postoperative Lymph Node Assessment in Patients with Oral Cancer

**DOI:** 10.2967/jnumed.121.262530

**Published:** 2022-05

**Authors:** Jasper Vonk, Jaron G. de Wit, Floris J. Voskuil, Yang Hang Tang, Wouter T.R. Hooghiemstra, Matthijs D. Linssen, Evert van den Broek, Jan J. Doff, Sebastiaan A.H.J. de Visscher, Kees-Pieter Schepman, Bert van der Vegt, Gooitzen M. van Dam, Max J.H. Witjes

**Affiliations:** 1 Department of Oral and Maxillofacial Surgery, University Medical Center Groningen, University of Groningen, Groningen, The Netherlands;; 2 Department of Pathology and Medical Biology, University Medical Center Groningen, University of Groningen, Groningen, The Netherlands;; 3 Department of Clinical Pharmacy and Pharmacology, University Medical Center Groningen, Groningen, The Netherlands;; 4 Department of Nuclear Medicine and Molecular Imaging and Medical Imaging Center, University Medical Center Groningen, University of Groningen, Groningen, The Netherlands; and; 5 TRACER BV, Groningen, The Netherlands

**Keywords:** fluorescence molecular imaging, lymph node metastasis, cetuximab-800CW, epidermal growth factor receptor, head and neck cancer

## Abstract

In most oral cancer patients, surgical treatment includes resection of the primary tumor combined with excision of lymph nodes (LNs), either for staging or for treatment. All LNs harvested during surgery require tissue processing and subsequent microscopic histopathologic assessment to determine the nodal stage. In this study, we investigated the use of the fluorescent tracer cetuximab-800CW to discriminate between tumor-positive and tumor-negative LNs before histopathologic examination. Here, we report a retrospective ad hoc analysis of a clinical trial designed to evaluate the resection margin in patients with oral squamous cell carcinoma (NCT02415881). **Methods:** Two days before surgery, patients were intravenously administered 75 mg of cetuximab followed by 15 mg of cetuximab-800CW, an epidermal growth factor receptor–targeting fluorescent tracer. Fluorescence images of excised, formalin-fixed LNs were obtained and correlated with histopathologic assessment. **Results:** Fluorescence molecular imaging of 514 LNs (61 pathologically positive nodes) could detect tumor-positive LNs ex vivo with 100% sensitivity and 86.8% specificity (area under the curve, 0.98). In this cohort, the number of LNs that required microscopic assessment was decreased by 77.4%, without missing any metastases. Additionally, in 7.5% of the LNs false-positive on fluorescence imaging, we identified metastases missed by standard histopathologic analysis. **Conclusion:** Our findings suggest that epidermal growth factor receptor–targeted fluorescence molecular imaging can aid in the detection of LN metastases in the ex vivo setting in oral cancer patients. This image-guided concept can improve the efficacy of postoperative LN examination and identify additional metastases, thus safeguarding appropriate postoperative therapy and potentially improving prognosis.

## Visual Abstract

**Figure:**
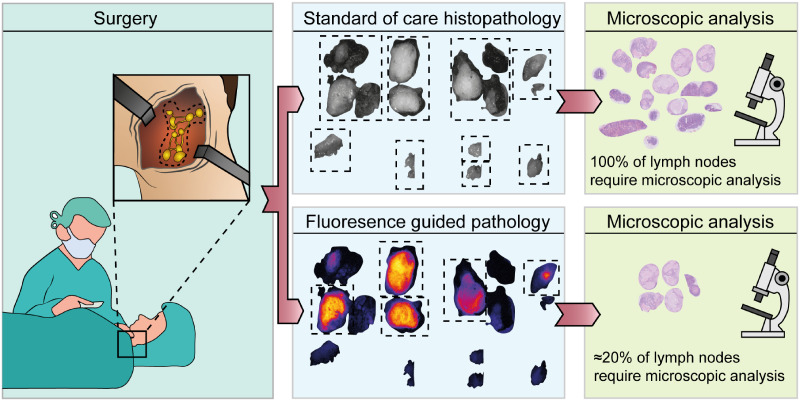

In oral squamous cell carcinoma (OSCC), the presence of lymph node (LN) metastasis has a major impact on prognosis and is associated with a significantly reduced survival (*1*,*2*). Consequently, assessment of LN status is important for determining the postoperative treatment strategy for the neck and consists of clinical assessment and preoperative radiographic imaging (i.e., MRI, CT, or ultrasound). If clinically suggestive LNs (cN+) are identified, a therapeutic neck dissection is indicated. However, even for a clinically node-negative neck (cN0), an elective neck dissection or sentinel node dissection is widely performed for staging because up to 30% of these patients have occult LN metastases or micrometastases (*3*,*4*). Postoperatively, the neck dissection specimen is macroscopically analyzed by the pathologist for the presence of LNs (*5*), and all LNs are sectioned and stained with hematoxylin and eosin or cytokeratin for microscopic evaluation. Other techniques for identifying LN metastasis are not clinically available yet. It is therefore interesting to explore other methods of identifying metastasis in LNs, especially when the tissue is intact, before routine processing.

Fluorescence molecular imaging (FMI), especially in the near-infrared window, is a rapidly evolving imaging technique in surgical oncology (*6*). FMI can provide real-time information on subsurface tissue by visualizing tumor-specific contrast agents (*7*), particularly when a controlled imaging environment is ensured (*8*). An interesting target for FMI is the epidermal growth factor receptor (EGFR), which is overexpressed in up to 90% of OSCC (*9*). Several phase I studies have shown the potential of EGFR-targeted FMI for intraoperative ex vivo tumor margin assessment in OSCC (*10*–*13*). However, little is known about EGFR-targeted imaging and identification of OSCC metastasis in LNs. FMI may allow for simultaneous ex vivo assessment of LN status when a neck dissection is performed together with removal of the primary tumor.

In this study, we explored the potential of FMI using cetuximab-800CW for discrimination between pathologically positive and negative LNs before histopathologic examination. The LNs were harvested as part of a clinical trial for resection margin assessment in OSCC patients (NCT03134846) (*10*).

## MATERIALS AND METHODS

### Clinical Trial Design

This prospective, cross-sectional, single-center diagnostic study was performed at the University Medical Center Groningen. The study was a retrospective ad hoc analysis of a clinical trial for resection margin evaluation (NCT02415881) (*10*). The clinical trial was approved by the Institutional Review Board of the University Medical Center Groningen (METc 2016/395) and was performed following the Dutch Act on Medical Research involving Medical Subjects and the Declaration of Helsinki (adapted version 2013). Written informed consent was obtained from all patients before any study-related procedures took place.

### Study Population

Patients with biopsy-confirmed OSCC who were scheduled for surgical removal of the tumor with concurrent neck dissection were eligible for inclusion in this study. Patients were excluded if they had a life expectancy of less than 12 wk, a Karnofsky performance status of less than 70%, a history of infusion reactions to monoclonal antibody therapies, QT prolongation on a screening electrocardiogram, uncontrolled medical conditions or episodes within 6 mo before enrollment (including uncontrolled hypertension, a cerebrovascular accident, or significant cardiopulmonary or liver disease), pregnancy, an abnormal electrolyte status, use of a class IA or III antiarrhythmic drug, or administration of an investigational drug within 30 d before the infusion of cetuximab-800CW.

### Synthesis of Cetuximab-800CW

Cetuximab-800CW was produced in the good-manufacturing-practice facility of the University Medical Center Groningen, as previously described (*14*). In short, cetuximab (Erbitux; ImClone LLC) was conjugated to IRDye800CW (LI-COR Biosciences Inc.) and purified using PD-10 desalting columns (Cytiva Life Sciences) under controlled conditions. Cetuximab-800CW was formulated in a sodium-phosphate buffer at a concentration of 1.0 mg/mL.

### Study Procedures

**FIGURE 1.**
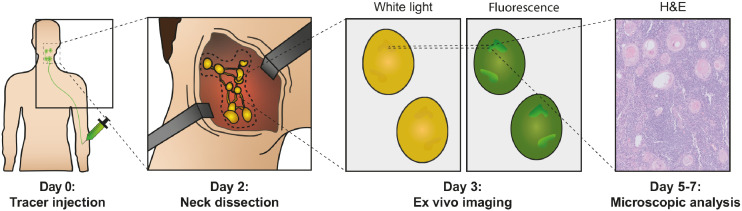
Summary of study workflow. All patients were administered fluorescent tracer cetuximab-800CW intravenously 2 d before surgery. After primary tumor surgery and neck dissection, nodal specimens were submitted to Department of Pathology and subsequently fixated in formalin for at least 24 h. All formalin-fixed tissue that could involve LNs was imaged in closed-field imaging system and underwent standard-of-care microscopic evaluation to correlate fluorescence signal with hematoxylin and eosin histopathology. H&E = hematoxylin and eosin.

The complete study workflow is summarized in Figure 1. Patients enrolled in the study received an unlabeled dose of 75 mg of cetuximab by slow infusion, followed by a bolus injection of 15 mg of cetuximab-800CW 2 d before surgery to ensure optimal visualization of the primary tumor (*10*). All patients underwent tumor surgery with concurrent neck dissection according to the standard of care. After surgery, neck dissection specimens were transferred to the Department of Pathology and formalin-fixed for at least 24 h. LNs were identified by visual and tactile inspection of the neck dissection specimen, were bisected when large enough, and subsequently were collected in cassettes. Single LNs were imaged in a closed-field fluorescence imaging system (Pearl Trilogy; LI-COR BioSciences) at the 800-nm channel, with the center cutting plane (i.e., inner side of the LN) faced toward the camera. Regions of interest were drawn around the entire tissue specimen included in the cassette, before microscopic assessment.

According to the standard of care, tissue was embedded in paraffin, and 4-µm tissue sections were cut from all formalin-fixed paraffin-embedded tissue blocks and then stained with hematoxylin and eosin. After routine tissue processing, we performed fluorescence flatbed scanning of these tissue blocks (Odyssey CLx; LI-COR Biosciences). EGFR immunohistochemistry was performed of LNs from patients harboring metastases to correlate fluorescence localization with histology. A head and neck pathologist, unaware of the results of FMI, analyzed all tissue sections for the presence of tumor cells and immunohistochemistry results.

### Statistical Analysis

Statistical analyses and graph designs were performed using Prism (version 9.0; GraphPad Software Inc.). Descriptive statistics were performed on patient demographics. The mean fluorescence intensities (FI_mean_) and maximal fluorescence intensities (FI_max_) of all LNs were calculated in ImageJ (Fiji, version 2.0.0) from the images obtained with the Pearl Trilogy. FI_mean_ was defined as total counts per region-of-interest pixel area (signal per pixel). FI_max_ was defined as the highest count measured within a region-of-interest pixel area. To improve the readability of this article, fluorescence intensities have been multiplied by 10^2^. Data were tested for a gaussian distribution using Shapiro–Wilk and Anderson–Darling tests; none of the data were normally distributed. We used the Mann–Whitney *U* test for statistical analysis of data; all data were unpaired. Correlations were measured using the Spearman rank correlation coefficient. Cutoffs were based on the Youden index. Data were presented as median with range or interquartile range (IQR). Statistical significance was determined as a *P* value of less than 0.05.

## RESULTS

Between January 2019 and February 2020, 22 patients were enrolled in this study. In total, 21 patients received the study drugs, consisting of an unlabeled dose of 75 mg of cetuximab followed by 15 mg of cetuximab-800CW, 2 d before surgery. One patient developed an adverse reaction during the unlabeled-cetuximab administration and was therefore excluded from the study. All remaining 21 patients completed the imaging protocol. The study procedures are summarized in Figure 1. Preoperative radiographic imaging (CT or MRI) was performed on all patients according to the standard of care. Thirteen (61.9%) of 21 patients were staged as cN0, 2 (9.5%) as cN1, 4 (19.0%) as cN2, and 2 (9.5%) as cN3. Five patients presented with extranodal extension. Fourteen elective neck dissections and 12 therapeutic neck dissections were performed, with 5 patients undergoing bilateral neck dissection. In total, 733 specimens considered to involve a LN were submitted for processing and subsequent microscopic analysis. Of these, 145 specimens were excluded because inking of the neck dissection specimen interfered with fluorescence imaging, resulting in a total of 588 specimens suitable for analysis. Of these, 514 included LNs based on final histopathology. The remaining 74 specimens contained no LNs. The number of LNs imaged after bisection was 239, whereas the number imaged intact was 275. Specimen and patient characteristics are shown in Table 1.

**TABLE 1:** Patient Demographics and Tumor Characteristics of All Patients

Characteristic	pN+ (*n* = 7)	pN− (*n* = 14)	All patients (*n* = 21)
Age (y)	67 (65–82)	64 (29–78)	66 (29–82)
Female	6 (85.8)	8 (57.1)	14 (67.7)
Weight (kg)	73 (52–105)	84 (53–140)	80 (52–140)
BSA (m^2^)	1.87 (1.52–2.17)	1.99 (1.58–2.67)	1.96 (1.52–2.67)
LNs	261	358	619
Level I	49 (18.8)	72 (20.1)	121 (19.5)
Level II	50 (19.2)	102 (28.5)	152 (24.6)
Level III	74 (28.4)	121 (33.8)	195 (31.5)
Level IV	58 (22.2)	47 (13.1)	105 (17.0)
Level V	30 (11.5)	16 (4.5)	46 (7.4)
Positive LNs^†^	64	NA	64
Level I	5 (7.8)		5 (7.8)
Level II	11 (17.2)		11 (17.2)
Level III	19 (29.7)		19 (29.7)
Level IV	19 (29.7)		19 (29.7)
Level V	10 (15.6)		10 (15.6)
Patients with ENE	5 (62.5)	NA	5 (23.8)
pN-stage*			
N0	0 (0)	14 (100)	14 (66.7)
N1	2 (28.6)	0	2 (9.5)
N2	4 (81.6)	0	4 (19.0)
N3	1 (20.4)	0	1 (4.8)
pT-stage			
T1	1 (14.3)	5 (35.7)	6 (28.6)
T2	2 (28.6)	3 (21.4)	5 (23.8)
T3	1 (4.8)	0	1 (4.8)
T4	3 (42.9)	6 (42.9)	9 (42.9)
Neck dissection^†^			
Elective	11 (64.7)	3 (33.3)	14 (53.8)
Therapeutic	6 (35.3)	6 (66.7)	12 (46.2)

*Initially, 6 patients were diagnosed with pathologically positive neck. Since 3 additional metastases were found on basis of FMI, total of 64 tumor-positive LNs was found, and 1 patient was upstaged from pN0 to pN1.

^†^Five patients received bilateral neck dissection, and total number of neck dissections therefore equals 26.

BSA = body surface area; ENE = extranodal extension.

Qualitative data are number and percentage; continuous data are median and range.

### Differentiation Between Pathologically Positive and Negative LNs

**FIGURE 2.**
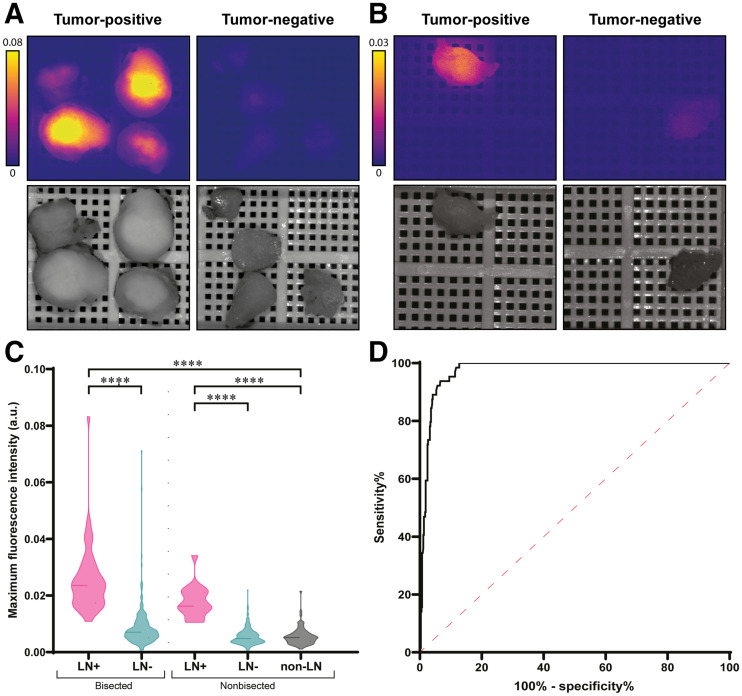
FMI with cetuximab-800CW enables discrimination between positive and negative LNs. (A and B) Representative images of bisected (A) and nonbisected (B) pathologically positive and negative formalin-fixed LNs from subject who was diagnosed with metastases on final histopathology. Increased fluorescence intensity was observed in both bisected and nonbisected pathologically positive LNs, compared with pathologically negative LNs. (C) FI_max_ is significantly increased in pathologically positive LNs, compared with negative LNs and non-LNs, both in bisected and in nonbisected LNs. (D) Receiver-operating-characteristic curve–based FI_max_ shows high area under curve of 0.98. ****P < 0.0001.

All specimens that were clinically considered as LNs (*n* = 588) were imaged after formalin fixation and before histopathologic examination. Six of 21 patients were diagnosed with LN metastasis on final histopathology, with a total of 61 pathologically positive LNs. Two parameters were measured during fluorescence imaging, FI_mean_ and FI_max_. At least a 3-fold increase in both FI_max_ and FI_mean_ was found in pathologically positive LNs (*n* = 61), compared with negative LNs (*n* = 453) or non-LN adipose or connective tissue (non-LNs) (*n* = 74) (Figs. 2A and 2B). The FI_max_ of pathologically positive LNs was 2.19 arbitrary units (a.u.) (IQR, 1.68–2.71 a.u.), compared with 0.57 a.u. (IQR, 0.39–0.80 a.u.) in negative LNs (*P* < 0.0001) and 0.51 a.u. (IQR, 0.36–0.65 a.u.) in non-LNs (*P* < 0.0001) (Fig. 2C). FI_mean_ was 0.92 a.u. (IQR, 0.73–1.20 a.u.) in pathologically positive LNs versus 0.22 a.u. (IQR, 0.14–0.33 a.u.) in negative LNs (*P* < 0.0001) and 0.21 a.u. (IQR, 0.13–0.32 a.u.) in non-LNs (*P* < 0.0001) (Supplemental Fig. 1A; supplemental materials are available at http://jnm.snmjournals.org).

### The Impact of LN Bisection on Fluorescence Intensity

During pathology processing, LNs were bisected if large enough and imaged with the center cutting plane (i.e., inner side of the LN) faced toward the camera. Bisected LNs showed a higher fluorescence intensity than did intact LNs (Figs. 2A and 2B; Supplemental Fig. 1A). Within pathologically positive LNs, bisected LNs (*n* = 44) showed an FI_max_ of 2.39 a.u. (IQR, 1.81–3.01 a.u.) and an FI_mean_ of 1.02 a.u. (IQR, 0.77–1.29 a.u.), compared with an FI_max_ of 1.63 a.u. (IQR, 1.42–2.12 a.u.) and an FI_mean_ of 0.78 a.u. (IQR, 0.62–0.93 a.u.) in nonbisected LNs (*n* = 17) (*P* = 0.0013 and 0.031, respectively). In pathologically negative LNs, bisected LNs (*n* = 195) showed an FI_max_ of 0.71 a.u. (IQR, 0.51–1.04 a.u.) and an FI_mean_ of 0.26 a.u. (IQR, 0.18–0.41 a.u.), compared with an FI_max_ of 0.48 a.u. (IQR, 0.36–0.68 a.u.) and an FI_mean_ of 0.19 a.u. (IQR, 0.12–0.29 a.u.) in nonbisected LNs (*n* = 258) (both *P* < 0.0001). In addition, body surface area showed a low correlation with FI_max_ (*R* = −0.44, *P* = 0.048) but not with FI_mean_ (*R* = −0.37, *P* = 0.103) in bisected pathologically negative LNs. The correlation between body surface area and FI_max_ (*R* = −0.64, *P* = 0.002) and FI_mean_ (*R* = −0.57, *P* = 0.011) was moderate in nonbisected pathologically negative LNs.

### The Impact of LN Size and Tumor Volume on Fluorescence Intensity

Topographic studies show that a metastatic tumor does not always involve the largest node within a neck dissection specimen (*15*), emphasizing the need to develop a tool that can also detect small metastases. First, to study the impact of LN size on fluorescence intensity, we correlated the diameter of pathologically negative LNs with both FI_max_ and FI_mean_. In all LNs (*n* = 514), a weak correlation was found between LN diameter and FI_max_ (*R* = 0.239, *P* < 0.0001) and FI_mean_ (*R* = 0.334, *P* < 0.0001). Subsequently, in pathologically positive LNs, we studied the impact of total tumor surface area and viable tumor surface area (i.e., total tumor surface area minus necrotic surface area) on fluorescence intensity. A moderate correlation was found between total tumor surface area and FI_max_ (*R* = 0.65, *P* < 0.0001) and FI_mean_ (*R* = 0.52, *P* < 0.0001). Viable tumor surface area also showed a moderate correlation with FI_max_ (*R* = 0.64, *P* < 0.0001) and FI_mean_ (*R* = 0.53, *P* < 0.0001).

### The Impact of FMI on Efficacy of LN Evaluation and Identification of Additional Metastases

Next, we evaluated whether FMI could discriminate between benign LNs and LNs containing metastasis. To mimic the clinical situation, we included all tissue fragments submitted to the pathologist (i.e., including non-LNs) in the analysis. On the basis of the Youden index, the cutoff rendered for FI_max_ was 1.048 a.u., resulting in 100% sensitivity, 86.8% specificity, a 48.9% positive predictive value, a 100% negative predictive value, and 88.2% accuracy, with an area under the curve of 0.98 (Table 2; Fig. 2D). As such, the FI_max_ cutoff allowed for a 77.4% decrease in LNs requiring microscopic examination without missing LN metastasis. For FI_mean_, the cutoff rendered was 0.508 a.u., resulting in 91.8% sensitivity, 91.9% specificity, a 59.6% positive predictive value, a 99.0% negative predictive value, and 91.9% accuracy, with an area under the curve of 0.98 (Table 2; Supplemental Fig. 1B). Receiver-operating-characteristic curves for both bisected and nonbisected LNs are provided in Supplemental Figure 2.

**TABLE 2:** Performance of Fluorescence Imaging Using Cetuximab-800CW at Optimal Cutoff for Selection of At-Risk LNs

Cutoff	Sensitivity (%)	Specificity (%)	PPV (%)	NPV (%)	Accuracy (%)	Preselected LNs (%)
FI_max_ ≥ 1.048	100.0%	86.8%	48.9%	100.0%	88.2%	22.6%
FI_mean_ ≥ 0.508	91.8%	91.9%	59.6%	99.0%	91.9%	17.2%

PPV = positive predictive value; NPV = negative predictive value.

Based on receiver-operating-characteristic curves, optimal fluorescence intensity cutoffs were determined to discriminate between positive LNs and negative LNs. Here, 100% sensitivity and NPV were applied as main criteria for use of FMI as selection tool for pathologist. Missing LN metastases should be avoided since appropriate postoperative therapy is essential to optimize prognosis.

Since FI_max_ resulted in a negative predictive value of 100%, a random sample of 40 false-positives based on FI_max_ (i.e., FI_max_ above the cutoff, pathologically tumor-negative) were additionally examined by serial sectioning according to the sentinel LN protocol to trace any missed metastases or micrometastases by the standard of care, as previously described (*4*). This random sample showed a median FI_max_ of 1.38 a.u. (IQR, 1.27–1.62 a.u.), compared with 1.34 a.u. (IQR, 1.17–1.65 a.u.) in the complete false-positive cohort (*P* = 0.35) and thus was considered a representative sample. Three additional positive LNs (7.5%) were identified in 2 patients. In both patients, the additional positive LNs resulted in upstaging of the neck from pN1 to pN2b. In 1 patient, this would have resulted in an intensified postoperative therapy, which, on the basis of standard-of-care histopathology, had not been performed.

### Microscopic Analysis of LNs

**FIGURE 3.**
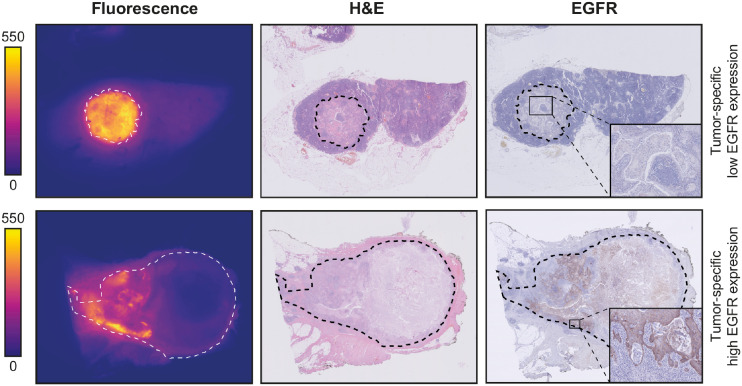
Microscopic analysis. Representative images of formalin-fixed LN metastases that were diagnosed on final histopathology. On both fluorescence images and hematoxylin- and eosin-stained slides, tumor region is delineated with dashed line. Fluorescence flatbed scanning shows increased fluorescence intensity in tumor deposits, compared with adjacent lymphoid and connective tissue. Although EGFR expression is variable within patients, fluorescence signal is tumor-specific, suggesting that other mechanisms play a role in cetuximab-800CW accumulation. H&E = hematoxylin and eosin.

To study the distribution of cetuximab-800CW at the microscopic level, EGFR immunohistochemistry was performed on a selection of pathologically positive and negative LNs. No EGFR expression was found in the negative LNs. In positive LNs, variable expression of EGFR was observed. Although EGFR expression colocalized with fluorescence signal, tumor regions without EGFR expression also showed high fluorescence, suggesting that a tumor-specific fluorescence signal is not mediated only by EGFR expression (Fig. 3).

To explain this observed fluorescence signal distribution, hematoxylin- and eosin-stained sections of pathologically positive LNs were further analyzed. Heterogeneous fluorescence intensities were observed between different tumor deposits. Within tumor deposits, we observed a higher fluorescence signal in the periphery of tumor deposits than in the center, as agrees with previous studies (*16*). Generally, we observed an increased fluorescence signal in regions with high tumor cell density and poor differentiation. Regions with abundant desmoplastic stroma or keratinization, associated with low cellularity, showed very low fluorescence intensities. Lastly, in necrotic areas, no fluorescence signal was observed.

Fluorescence false-positive LNs were examined microscopically. As mentioned before, 3 (7.5%) additional metastases were detected. In other fluorescence false positives, we consistently found high vascularization compared with true-negative LNs, specifically colocalizing with areas showing a high fluorescence intensity at fluorescence flatbed scanning.

## DISCUSSION

This study demonstrated that EGFR-targeted FMI based on intravenously administered cetuximab-800CW can be used to discriminate pathologically positive LNs from negative LNs. A cutoff of 1.048 a.u. for FI_max_ resulted in the detection of positive LNs with 100% sensitivity and a 100% negative predictive value. Therefore, FMI can safely reduce the number of LNs requiring histopathologic examination by 77.4% and improve the efficiency of pathology processing without missing any metastases. Importantly, FMI detected pathologically positive LNs in 7.5% of the LNs initially false-positive on fluorescence imaging, which were missed by standard-of-care histopathology. Because the pathologic stage of the neck often drives recommendations for the postoperative therapy strategy, these missed positive LNs could have a major impact on the adequacy of postoperative treatment and, therefore, prognosis.

The use of EGFR-targeted FMI for the detection of LN metastasis in OSCC patients before formalin fixation has previously been evaluated (*17*–*19*). In dose-escalation studies with cetuximab-800CW and panitumumab-800CW, tumor-positive LNs were identified with high sensitivity, although a dose-dependent increase in falsely fluorescence-positive LNs was observed (*18*,*19*). One study showed that signal-to-noise ratio and FI_mean_ could guide the ex vivo assessment of nodal specimens and identify tumor-positive LNs with high sensitivity and specificity (*18*). Yet, because the use of signal-to-background ratio requires knowledge of the presence and dimensions of a possible tumor, this strategy cannot be applied to select at-risk LNs before histopathologic evaluation. More recently, Krishnan et al. reported the administration of 50 mg of panitumumab-800CW 1–5 d before surgery for LN assessment. When analyzing the top 5 LNs, the authors found that use of a fluorescence nodal ranking method achieved accurate nodal staging in all patients (*17*). Because this method is based on relative fluorescence intensities, microscopic examination of LNs is required in all patients, even when low absolute fluorescence intensities are observed. Since most patients (55.6% in their cohort) have a pathologically negative neck, we believe that using an FI_max_ cutoff is favorable in that it rules out the need to examine LNs microscopically in all patients.

**FIGURE 4.**
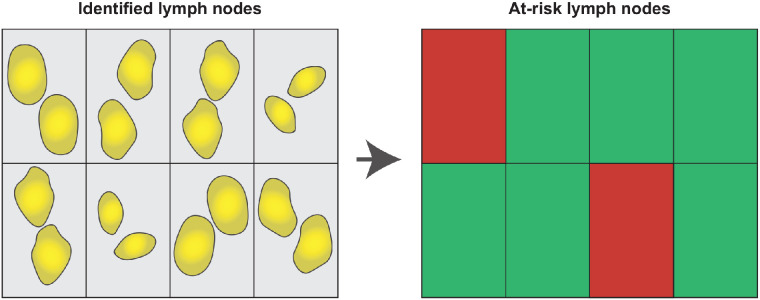
Grid selection of LNs for microscopic evaluation. Using grid, fluorescence imaging of identified LNs can automatically identify LNs that display FI_max_ above cutoff. In contrast to FI_mean_, this approach does not require drawing region of interest around LNs. As such, at-risk LNs can be selected rapidly without interfering with standard of care.

The uniqueness of our data, compared with the previous studies described, lies in the consistent administration of a single dose of cetuximab-800CW 2 d before surgery, allowing us to propagate a reliable cutoff for subsequent studies. We advocate the use of FI_max_ over FI_mean_ for swift clinical implementation since it does not require additional steps between imaging and selection, such as the drawing of regions of interest. Here, we propose using a grid to automatically identify LNs on the basis of the FI_max_ measured in each square of the grid (Fig. 4). This method enables user-friendly evaluation of all harvested LNs within minutes while reducing the LNs requiring microscopic assessment by 78.0%.

Although these results are promising for clinical use, our study had some limitations. Despite the use of a low dose of cetuximab-800CW, which empirically would decrease the number of false-positives (*18*,*19*), our dosing strategy was optimized for assessing the margin of the primary tumor rather than for evaluating LNs. Second, although we observed a high fluorescence intensity in all regions showing EGFR expression, high fluorescence intensities were also found in regions without EGFR expression. This varying colocalization was also described earlier by Nishio et al. (*18*) and coincides with studies finding that EGFR expression did not correlate with cetuximab uptake in PET imaging (*20*,*21*) and could not predict the response to cetuximab therapy (*22*–*24*). The significance of EGFR staining is questionable, as it has been shown that EGFR expression as determined by immunohistochemistry is not the sole reflection of tumor biology (*25*).

As such, we hypothesize that additional mechanisms within the tumor microenvironment influence the accumulation of cetuximab-800CW and that the presence of EGFR may not be the only determinant, as has also been observed in EGFR-targeted photodynamic therapy (*26*,*27*). Multiple studies on FMI and other imaging modalities have pointed out the role of vascularization and interstitial pressure in the accumulation of targeted contrast agents (*16*,*20*,*24*). This possible role does also fit the observation that the fluorescence false-positives in the current study showed aberrant vascularization, possibly leading to the accumulation of cetuximab-800CW through the effect of enhanced permeability and retention.

In future, studies could evaluate new dosing strategies dedicated to the assessment of LNs, such as adding a second, untargeted, tracer with different spectral properties. This strategy would enable correction for nonspecific tracer accumulation and increase the contrast between tumor tissue and nontumor tissue within LNs (*28*). This enhanced contrast may further increase the accuracy of FMI for postoperative LN assessment. Second, we hypothesize that these FMI results can be translated to the assessment of freshly excised LN specimens, albeit fresh LNs may show slightly different fluorescence intensities as no formalin fixation is performed before imaging. This difference may impact the signal because of washout of nonspecific fluorescent tracer or alteration of tissue optical properties (*29*,*30*). Here, the intraoperative use of FMI depends on the surgical procedure performed on the neck. Intraoperative LN biopsies allow for immediate intraoperative imaging since single LNs are excised. However, FMI could also be used for the analysis of an elective neck dissection specimen, although this use is logistically more challenging since it requires intraoperative fluorescence analysis of LNs by a second clinician (e.g., a pathologist or a lab technician). Intraoperative identification of a tumor-positive LN enables direct extension to a therapeutic neck dissection if possible (*13*), which may prevent a second surgery and eventually will decrease both patient burden and health-care costs by reducing operation time.

## CONCLUSION

Our findings suggest that FMI with the intravenously administered EGFR-targeting fluorescent tracer cetuximab-800CW can aid in the detection of LN metastases in the ex vivo setting in OSCC patients. We demonstrated that this method could improve the efficiency of postoperative LN assessment without missing LN metastases. Importantly, FMI may identify additional LN metastases, leading to more accurate staging of the neck and appropriate postoperative treatment, which may eventually improve prognosis.

## DISCLOSURE

This research was funded by the Dutch National Cancer Society (RUG 2015-8084). Gooitzen van Dam is the CEO, the founder, and a shareholder of TRACER Europe BV/AxelaRx. Bert van der Vegt is a member of the Scientific Advisory Board of Visiopharm, for which compensation is received by the University Medical Center Groningen. No other potential conflict of interest relevant to this article was reported.

## Notes

KEY POINTS **QUESTION:** Can EGFR-targeted FMI differentiate between tumor-positive and tumor-negative LNs? **PERTINENT FINDINGS:** In this retrospective ad hoc analysis, we showed that preoperative intravenously administered cetuximab-800CW could detect tumor-positive LNs ex vivo with 100% sensitivity and 86.8% specificity (area under the curve, 0.98). Additionally, in 7.5% of the 38 LNs false-positive on fluorescence imaging, we identified additional metastases missed by the standard of care. **IMPLICATIONS FOR PATIENT CARE:** This image-guided concept may improve the efficacy of LN processing while detecting additional metastases, thus safeguarding appropriate postoperative therapy and potentially improving the prognosis.
